# Exploring the Role of the *MUTYH* Gene in Breast, Ovarian and Endometrial Cancer

**DOI:** 10.3390/genes15050554

**Published:** 2024-04-26

**Authors:** Carla Lintas, Benedetta Canalis, Alessia Azzarà, Giovanna Sabarese, Giuseppe Perrone, Fiorella Gurrieri

**Affiliations:** 1 Research Unit of Medical Genetics, Department of Medicine, University Campus-Biomedico of Rome, Via Alvaro del Portillo 21, 00128 Roma, Italy; f.gurrieri@unicampus.it; 2 Operative Research Unit of Medical Genetics, Fondazione Policlinico Universitario Campus Bio-Medico, Via Alvaro del Portillo 200, 00128 Roma, Italy; a.azzara@unicampus.it; 3 Operative Research Unit of Anatomical Pathology, Fondazione Policlinico Universitario Campus Bio-Medico, Via Alvaro del Portillo 200, 00128 Roma, Italy; benedetta.canalis@policlinicocampus.it (B.C.); g.sabarese@policlinicocampus.it (G.S.); g.perrone@policlinicocampus.it (G.P.); 4 Research Unit of Anatomical Pathology, Department of Medicine, Università Campus Bio-Medico di Roma, Via Alvaro del Portillo 21, 00128 Roma, Italy

**Keywords:** breast cancer, *MUTYH*, second hit, germinal, autosomal recessive, autosomal dominant

## Abstract

Background: *MUTYH* germline monoallelic variants have been detected in a number of patients affected by breast/ovarian cancer or endometrial cancer, suggesting a potential susceptibility role, though their significance remains elusive since the disease mechanism is normally recessive. Hence, the aim of this research was to explore the hypothesis that a second hit could have arisen in the other allele in the tumor tissue. Methods: we used Sanger sequencing and immunohistochemistry to search for a second *MUTYH* variant in the tumoral DNA and to assess protein expression, respectively. Results: we detected one variant of unknown significance, one variant with conflicting interpretation of pathogenicity and three benign/likely benign variants; the *MUTYH* protein was not detected in the tumor tissue of half of the patients, and in others, its expression was reduced. Conclusions: our results fail to demonstrate that germinal monoallelic *MUTYH* variants increase cancer risk through a LOH (loss of heterozygosity) mechanism in the somatic tissue; however, the absence or partial loss of the *MUTYH* protein in many tumors suggests its dysregulation regardless of *MUTYH* genetic status.

## 1. Introduction

Autosomal recessive familial adenomatous polyposis-2 (FAP2) (OMIM *604933) is caused by germline biallelic variants in *MUTYH*, a gene whose product is involved in the base excision repair (BER) mechanism. The *MUTYH*-encoded protein protects DNA from reactive oxygen species, methylation, deamination, hydroxylation and other products of cellular metabolism [1]. Specifically, *MUTYH* is a DNA glycosylase that protects the cell from guanine oxidation by removing the modified base. The gene is expressed in the mitochondria and the nuclei of human cells [2]. The *MUTYH* gene acts as an oncosuppressor and acts in a recessive way, as two biallelic variants or one homozygous variant are required to inactivate its function. *MUTYH* missense variants, frameshift and stop variants have been reported. In the ClinVar database (https://www.ncbi.nlm.nih.gov/clinvar, accessed on 30 March 2024) 228 pathogenetic variants and 74 likely pathogenetic variants have been reported to date. Furthermore, epigenetic silencing with DNA methylation and enrichment of methylated histone signs are other reported mechanisms for the loss of heterozygosity (LOH) of the *MUTYH* gene [3].

With the recent advent of next-generation sequencing and the consequent introduction of multigene panel testing, the diagnostic yield in hereditary cancers has increased in the last few years, and new cancer-predisposing genes have emerged. *MUTYH* is usually included in such gene panels, and pathogenic variants are being increasingly detected in a heterozygous state in a consistent number of patients with different cancer types.

Among cancers, breast and ovarian neoplasms are the most common types in the female population, and about 10–15% of cases are familial. The diagnostic process of women affected by breast cancer on a hereditary basis involves a first-level analysis of *BRCA1* and *BRCA2* genes and, if negative and clinically indicated, a second-level analysis of other cancer genes, including *MUTYH*. Indeed, monoallelic germline *MUTYH* variants have been recurrently detected in patients affected by breast/ovarian cancer [4], as well as in patients with prostate cancer, endometrial and gastrointestinal cancers, including Lynch syndrome [5]. Lynch syndrome is an autosomal dominant condition that increases the cancer risk for the colon, uterus, urinary tract, kidneys, prostate, breast, liver, skin and brain. In this condition, a defective function of the mismatch repair (MMR) machinery has been established as the main pathogenetic cause. An MMR defect, related to somatic hypermethylation of the MLH1 gene, also occurs in non-Lynch neoplasms, indicating that MMR defects can be frequently involved in cancer development (especially of the colon and uterus), independently of a somatic or germinal genetic alteration. Even though recent reports suggest that monoallelic variants in *MUTYH* may increase the risk for cancer [4,5], their clinical significance is still unclear, as sufficient information on the pathogenic mechanism is lacking. However, the National Comprehensive Cancer Network guidelines (2022) recommend that individuals with monoallelic/MUTYH heterozygote variants have a colonoscopy every 5 years, starting from 40 years of age, or 10 years younger than a first-degree relative’s age at diagnosis of CRC (colorectal cancer).

As part of our routine diagnostic work-up, we have detected *MUTYH* germline heterozygous variants in a number of patients affected by breast/ovarian cancer or endometrial cancer who underwent a targeted gene panel analysis, as requested by the clinical geneticist. With the aim of understanding the clinical significance of these findings and defining the plausible molecular mechanism of pathogenicity, we hereby explore the hypothesis that a second hit could have arisen in the second allele in the tumor tissue, leading to a complete loss of function of this gene. Along with the somatic genetic testing, we also performed immunohistochemistry targeting *MUTYH* protein expression on the same tumoral tissue sections. Our cohort consisted of nine women (aged 26–80 years) affected by breast/ovarian cancer or endometrial cancer who underwent surgery in our hospital. In all these patients, a hereditary form of cancer was suspected based on their personal (young age/multiple neoplasia) and/or familial history.

## 2. Materials and Methods

### 2.1. Germline DNA Testing

Nine women aged between 26–80 years who had a diagnosis of breast/ovarian cancer or endometrial cancer were enrolled in this study. Clinical and demographic data are reported in Table 1. All women underwent tumor surgery in our hospital. Because of either the young age of cancer onset or their personal and familial history suggestive of inherited cancer predisposition, they all were considered eligible for germline genetic testing upon genetic counseling. In detail, at least one of the following criteria was satisfied: young age of cancer onset (before 40 years old), first-degree relatives affected by cancer, and the presence of bilateral breast cancer or multiple primary tumors. Patients affected by breast/ovarian cancer (Table 1) were all negative for pathogenetic/likely pathogenetic *BRCA1* and *BRCA2* variants (both single nucleotide variants—SNV and copy number variants—CNV). Similarly, patients affected by endometrial cancer (Table 1) were negative for pathogenetic/likely pathogenetic variants in the mismatch repair genes (*MLH1*, *MSH2*, *MSH6*, *PMS2*).

As a second-level analysis, a multigene panel was performed for all nine patients. The multigene panel included the following genes: *APC*, *ATM*, *AXIN2*, *BMPR1A*, *BRCA1*, *BRCA2*, *CDH1*, *CHEK2*, *DICER1*, *EPCAM*, *KIT*, *MLH1*, *MSH2*, *MSH3*, *MSH6*, *MUTYH*, *NF1*, *NTHL1*, *PDGFRA*, *PMS2*, *POLD1*, *POLE*, *PTEN*, *SDHB*, *SDHC*, *SMAD4*, *STK11*.

Upon informed consent, DNA was extracted from a peripheral blood sample, and NGS sequencing was performed as previously reported [6]. NGS sequencing was performed with an average coverage of 60× on an Illumina platform. The bioinformatic analysis was performed on the online platform Galaxy: the fastQ files were aligned using the Burrows–Wheeler Aligner [7] (Human GRCh38/hg19); duplicates were removed to perform the variant calling with FreeBayes and the variant annotation with wAnnovar.

All bioinformatic analyses were conducted following best practice recommendations. We included in our analysis all variants identified within the coding (missense, stop, frameshift and indels) and the splicing regions with good coverage. We also filtered for variants with a population frequency in the GnomAD database (https://gnomad.broadinstitute.org/, accessed on 30 March 2024) lower than 1%.

### 2.2. Somatic DNA Testing

Genomic DNA was isolated from formalin-fixed and paraffin-embedded (FFPE) tumoral tissue sections using the MagCore^®^ Genomic DNA FFPE One-Step Kit (RBC Bioscience Corp., New Taipei City, Taiwan), according to the manufacturer’s protocol. The concentration of total DNA was determined using the BioSpectometer (Eppendorf, Hamburg, Germany).

The coding region of *MUTYH* consisting of 16 exons was divided into nine amplicons (Supplementary Table S1) and PCR-amplified. The PCR conditions were as follows: (1) denaturation—5 min at 95 °C, (2) cycling for 35 cycles—30 s at 95 °C, 30 s at 60 °C, 30 s at 72 °C, (3) final extension at 72 °C for 10 min. PCR products were Sanger-sequenced. Sanger sequencing was used to sequence *MUTYH* PCR products in the somatic samples. Primers flanking the *MUTYH* exon variants were designed using Primer3 application on the UCSC genome browser. Using manufacturers’ guidelines, PCR products were cleaned up using a mixture of Exonuclease I and Shrimp Alkaline Phosphatase (ArticZymes, Tromsø, Norway), sequenced using BigDye terminator Kit (Applied Biosystems, Foster City, CA, USA) and run on a 3500xl Genetic Analyzer (Applied Biosystems, Foster City, CA, USA). The electropherograms were analyzed using Sequencing Analysis Software (v.6.0, Applied Biosystems, Foster City, CA, USA). Variants were annotated using bioinformatics tools, such as Varsome (https://varsome.com/, accessed on 30 March 2024) GnomAD (https://gnomad.broadinstitute.org/, accessed on 30 March 2024), ClinVar (https://www.ncbi.nlm.nih.gov/clinvar/, accessed on 30 March 2024), and in silico predictors such as Polyphen, Mutation Taster, Sift and others. These last bioinformatics tools are used to predict if missense variants can affect the normal protein function. In addition, to predict the functional consequences of variants that could potentially affect splicing, the database SpliceAI (https://spliceailookup.broadinstitute.org/, accessed on 30 March 2024) was used. The database GnomAD was instead used to evaluate the frequency of the variants in the general healthy population. Finally, the above-mentioned database ClinVar was used to see if the detected variants had been previously reported by other laboratories, Variants were, finally, classified according to the American College of Medical Genetics (ACMG) guidelines [7].

### 2.3. MUTYH Immunohistochemistry

Representative tumor blocks were sectioned at 3 mm thickness. Immunohistochemistry (IHC) was performed with the Anti-*MUTYH* rabbit polyclonal antibody PA5-75316 (Thermo Fisher Scientific, Waltham, MA, USA) at a 1:50 dilution.

Immunohistochemical staining was performed using the Ventana BenchMark Ultra immunostainer (Roche Diagnostics, Indianapolis, IN, USA) with the ultraView Universal DAB Detection Kit (Roche Diagnostics, Indianapolis, IN, USA).

The protein expression was evaluated both on cancer and non-cancer tissue. Two breast tumoral tissues derived from two *MUTYH* negative patients (patients without germinal *MUTYH* variants) were used as controls. Additional controls included non-cancer tissue such as tonsillae.

**Figure 1:**
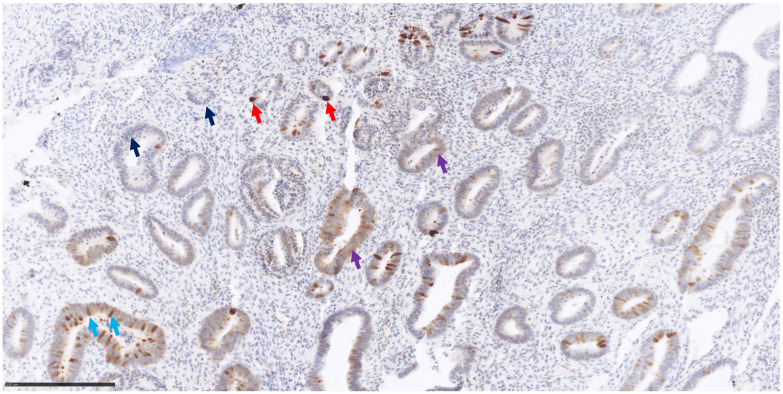
*MUTYH* immunohistochemistry staining and IHC score system. This field shows a heterogeneous expression of *MUTYH* protein: neoplastic cells negative for *MUTYH* expression defined as score 0 (blue arrows); neoplastic cells with weak positivity defined as score 1+ (purple arrows); neoplastic cells with moderate positivity scored as 2+ (light blue arrows); neoplastic cells with strong positivity scored as 3+ (red arrows). Breast/endometrial/ovarian cancer tissues. Magnification 200×.

The histochemical score (IHC score) was determined by evaluating the staining intensity and percentage of stained neoplastic cells at each intensity level (score 0, score 1+, score 2+, score 3+). In particular, the score 0 was assigned for negative staining of the neoplastic cells, a score of 1+ for weak staining of the neoplastic cells, a score of 2+ for moderate staining of the neoplastic cells and a score of 3+ for strong staining of the neoplastic cells (Figure 1).

The IHC score was calculated with the following formula:

IHC score = (0 × % score 0) + (1 × % score 1) + (2 × % score 2) + (3 × % score 3)

The values of each score vary from 0% to 100%. The IHC score, therefore, is in the range of from 0 to 300 [8]. IHC staining was independently evaluated by two pathologists (GP, GS), followed by a consensus session for discordantly scored samples to define a consensus score for each case. The pathologists were blinded to the germline DNA testing.

## 3. Results

### 3.1. MUTYH Germline Variants

Eight patients had *MUTYH* heterozygous pathogenetic variants (Table 2, panel A): four patients had the c.1187 G>A p.Gly396Asp variant, three patients had the c.536 A>G p. Tyr179Cys variant and one patient had the c.1437_1439del p.Glu480del. These three variants were all reported in the ClinVar database. The ninth patient, patient 8, had two different heterozygous variants, both of unknown clinical significance (VUS, variant of unknown significance): c.1258 C>A p. Leu420Met and c.694 A>T p.Thr232Ser variants. Segregation analysis showed that the two variants were in cis. Four patients had additional VUS variants in other genes as reported in Table 2, panel A. Patient 2 had a *BRCA1* variant (c.5332 G>A p.Asp1178Asn); patient 5 had *BRCA2* (c.3680 T>C p.Leu1227Pro) and *CDH1* (c.74C>T p.Pro25Leu) variants; patient 7 had *PALB2* (c.398 G>A p.Ser133Asn) and *ATM* (c.1516 G>T p.Gly506Cys) variants; patient 9 had a *POLE* variant (c.6445 C>T p.Arg2149Cys).

### 3.2. MUTYH Somatic Variants

As expected, all the constitutional variants were also detected in the tumoral DNA. In five out of nine patients, additional *MUTYH* variants were detected in the tumoral DNA (Table 2, panel B). Patient 8 and patient 9 share the same common variant (frequency in the GnomAD database equal to 0.286): c.1014 G>C p.Glu338His, which can be classified as benign according to the American College of Medical Genetics (ACMG) guidelines. Similarly, patient 5 and patient 7 have two different synonymous likely benign variants: c.312 C>T, p.Tyr104Tyr and c. 420 C>T, p. Thr140Thr, respectively. Patient 3 is the only patient carrying two somatic missense variants: c.770 T>C p.Leu257Pro and c.1256 C>T p.Ala419Val in exon 9 and exon 13, respectively (Table 2, panel B). Both variants are classified as *VUS* according to the ACMG guidelines. The first variant is absent from the population database GnomAD, whereas the second one is very rare, having a frequency equal to 0.00003184; in silico tools are not conclusive about a possible deleterious functional role for both variants (Table 2, panel B).

### 3.3. MUTHY Immunohistochemistry

**Figure 2:**
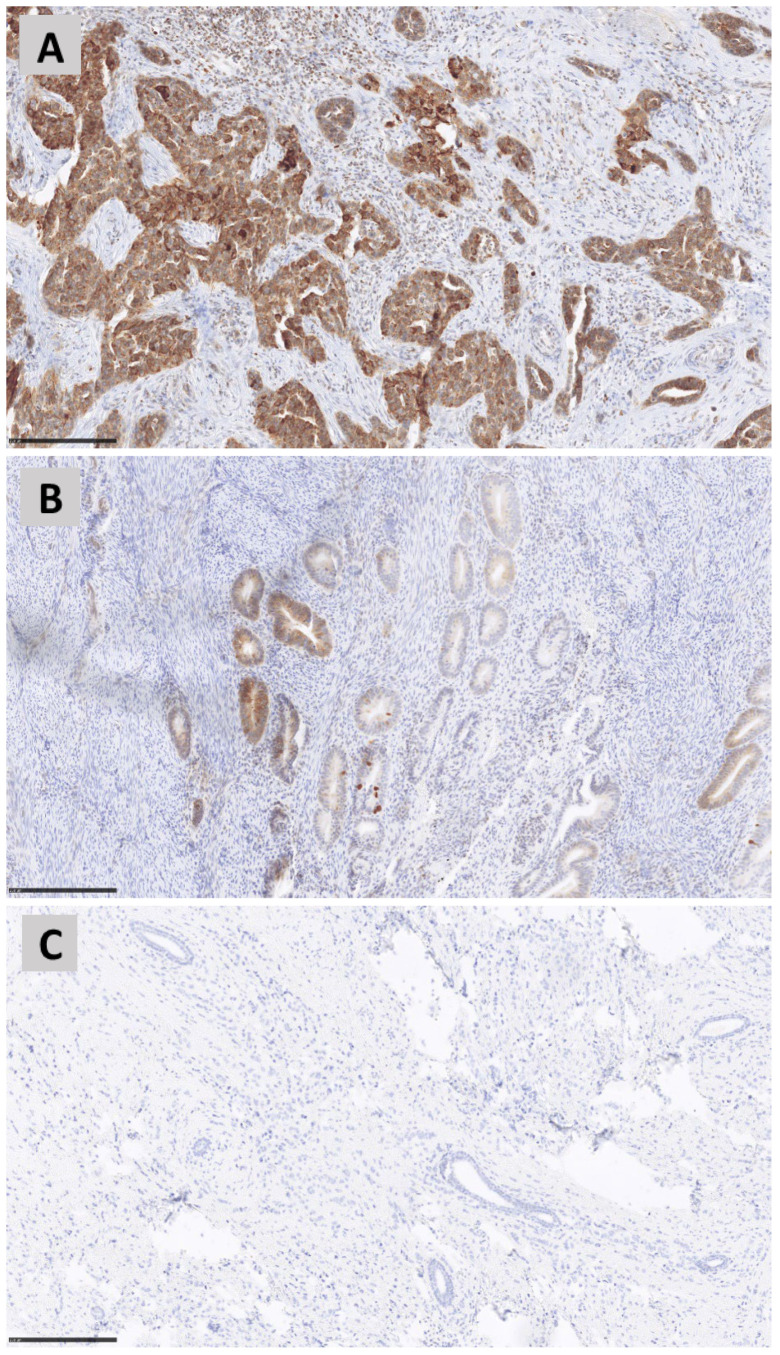
*MUTYH* immunohistochemical scoring system. Breast/endometrial/ovarian cancers show different staining patterns of *MUTYH* expression. Panel (**A**) represents breast cancer with diffuse (90% neoplastic cell) and strong staining intensity (score 3+). Panel (**B**), breast cancer with heterogeneous *MUTYH* expression: almost 40% of cancer cells show *MUTYH* positivity (score 2+), while for the rest, the result was negative (score 0). Panel (**C**), breast cancer completely negative for *MUTYH* staining (100% of neoplastic cell with score 0).

IHC results are reported in Table 3 and Figure 1 and Figure 2. *MUTYH* expression was detected in neoplastic cells with variable intensity (IHC score from 70 to 215 in pz 1, 3, 6, 7 and 9) in five out of nine patients carrying the *MUTYH* germinal variant (Figure 1 and Figure 2). Patient 7 had the highest *MUTYH* expression (total IHC score equal to 215), followed by patient 9 (total IHC score equal to 90), patient 3 (total IHC score equal to 80), patient 6 (total IHC score equal to 75) and patient 1 (total IHC score equal to 70). No expression was detected in the neoplastic cells of four patients (patient 2, patient 4, patient 5 and patient 8) carrying *MUTYH* variants and of the two tumoral control samples (Table 3, Figure 2, panel C) without *MUTYH* germinal variants. *MUTYH* protein expression (Supplementary Figure S1) was detected in non-neoplastic cells of all patients (lymphocytes), as well as in the nontumoral control sample (tonsillae). These last tissues and/or cells can be considered positive controls for IHC.

## 4. Discussion

*MUTYH*-associated polyposis is a well-known autosomal recessive condition due to pathogenic biallelic variants which increase the risk for polyposis, colorectal cancer and, to a smaller extent, other types of cancers. In a very recent study, Mak et al. [9] reported a 25.5% diagnostic yield for *MUTYH* biallelic mutations and *APC* monoallelic variants in a cohort of 259 patients with multiple colorectal adenomas. Immunohistochemistry studies have shown that in the normal colorectal tissue of healthy individuals, *MUTYH* expression is strong in the nuclei and weak in the cytoplasm [2]. On the other hand, in the normal and tumoral colon tissue of a patient affected by colon cancer (not carrying any *MUTYH* variants), expression is similar and is localized in both the nuclei and the cytoplasm. A third expression pattern is seen in the colorectal carcinoma or adenoma of patients carrying biallelic *MUTYH* variants: nuclear expression is absent, whereas cytoplasmatic expression is strong both in the neoplastic and surrounding healthy mucosa [10]. However, another paper on *MUTYH*-associated polyposis and immunostaining did not confirm such a finding showing strong *MUTYH* cytoplasmatic expression in the tumoral tissue of non-*MUTYH* carriers, suggesting that immunohistochemistry cannot be used to discriminate *MUTYH*-mutated from unmutated cases [11]. Along the same line, IHC results in our cohort appear not to be correlated with *MUTYH* variants (both germline and somatic). The lack of expression in the neoplastic cells of two control samples and of four tumoral cases could be due to either the specific tissue sections used for IHC analysis or to other unknown and not *MUTYH*-related biological factors (Table 3, Figure 1 and Figure 2). Indeed, to date, *MUTYH* expression has always been detected in both tumoral and normal tissues [2,10,11], and complete loss of function has never been documented in the literature. Furthermore, no correlation could be seen between the IHC score and *MUTYH* variants in the other five patients (positive for *MUTYH* expression) as all of them had one monoallelic pathogenetic *MUTYH* variant and no somatic variants of clinical significance. Additional variants in other *MUTYH*-interacting genes could account for such a discrepancy.

The recent introduction of multigene panels in cancer genetics has led to the detection of monoallelic germinal *MUTYH* variants in several types of neoplasms as breast, ovarian, endometrial and colorectal cancer. Less frequently, monoallelic *MUTYH* variants have also been detected in other types of cancer, such as gastric, lung, liver, biliary and small intestine tumors. In a recent report [12], the *MUTYH* variant c.1187 G>A (p. Gly396Asp) was found in a patient affected by melanoma with a family history of cancer. Nunziato et al. [4] screened with a multigene panel a cohort of 64 breast/ovarian cancer patients who tested negative for BRCA1/2 screening and with a positive personal and familial history of cancer. They found that the most mutated gene was *MUTYH* and reported three monoallelic pathogenetic/likely pathogenetic variants: one splicing variant (c.849 + 3 A>C) and two missense variants (c.1103 G>A. p.Gly368Asp and c.452 A>G p.Tyr151Cys). Monoallelic pathogenic *MUTYH* variants were also reported in patients affected by prostate cancer, with a frequency of about 0.5–1% [5,13]. In a study of 152 glioma patients [14], germline pathogenic *MUTYH* variants were detected in about 0.5% of patients. A recent study by Keske et al. [15] highlights how the clinical–pathological features of breast tumor patients carrying germline monoallelic *MUTYH* variants overlap with those carrying *BRCA1* germline variants. Specific and more aggressive histological features include larger tumor size, higher tumor grade and more high-risk biomarker profiles (Her2 positive and triple negative). They also found that about 50% of *MUTYH* heterozygous carriers had nonfamilial breast cancer, suggesting that the gene in those cases is not a major risk factor for breast cancer development, as instead reported for colorectal cancers [16,17].

However, the clinical significance of such findings remains elusive, given the known recessive mechanism of *MUTYH.* We wanted to explore whether a second *MUTYH* hit in the tumor genome would be necessary for cancer development. To this purpose, we have searched for the second hit in the tumoral DNA of nine patients affected by several types of cancer who carried a pathogenic germinal monoallelic *MUTYH* variant.

Our pilot study has shown that a second somatic *MUTYH* hit is an unlikely pathogenetic mechanism in monoallelic carriers with breast, ovarian and endometrial cancer. Indeed, we found somatic *MUTYH* variants only in five patients, and most of them were classified as benign, being either synonymous variants or common variants in the general population. Only patient 3 had two missense VUSs (variants of unknown significance) *MUTYH* somatic variants: c.770 T>C p.Leu257Pro and c.1256 C>T p. Ala419Val, which were absent and rare in the general population, respectively (Table 2, panel A). For both variants, the in silico predictor software was in conflict, with a prevalence of bioinformatic tools towards a benign effect. Patient 3 had endometrial cancer when she was 37 years old; she also had a strong positive cancer family history, having a sister affected by endometrial adenocarcinoma. Furthermore, both sisters lacked expression of *MSH2* and *MSH6* in the tumoral DNA, and no germinal single nucleotide variants in these two genes were detected. We cannot establish at the moment a correlation between the lack of protein expression of these two genes and the two identified VUS *MUTYH* variants.

Additionally, we noted that the most frequent pathogenic germinal variants detected in our patients were c.1187 G>A p.Gly396Asp and c. 536 A>G p.Tyr179Cys, which were present in four and three patients, respectively. These variants have been found to be relatively common in the general healthy European population, being present at a frequency of about 0.3% and 0.15%, respectively. Indeed, a recent study by Thomson et al. [18] on large case-control datasets has shown that the frequency of these two common variants classified in the clinical database ClinVar as pathogenetic is not significantly different between the cancer-free population and patients affected by colorectal, endometrial and breast cancer. A similar outcome was also observed for other *MUTYH* pathogenetic variants in the same different cancer type control cohorts. Therefore, this study would support a nonpathogenic role for monoallelic *MUTYH* variants in cancer pathogenesis.

The main limitation of our study is the small sample size. However, we would like to increase the sample size of our cohort by enrolling new patients as soon as they become available. Another important limitation of our study is that we perform only Sanger sequencing on the coding exons of *MUTYH* somatic DNA. This technique does not allow the detection of copy number loss and/or gene promoter methylation, which both represent common mechanisms for LOH (loss of heterozygosity) in tumor suppressor genes like *MUTYH*. Indeed, some authors [19,20] detected LOH of *MUTYH* due to copy number loss in the tumor of two breast cancer patients who had a first germline *MUTYH* pathogenetic variant. The authors [19] concluded that germline monoallelic inactivation of *MUTYH* is not sufficient for C:G>A:T transversion somatic signatures previously correlated with *MUTYH* deficiency to arise but that biallelic complete loss of function of the *MUTYH* gene is necessary to cause tumors similar to those associated with polyposis due to biallelic mutations in the *MUTYH* gene.

## 5. Conclusions

Our results fail to demonstrate that germinal monoallelic *MUTYH* variants increase cancer risk development through an LOH mechanism; however, IHC data demonstrate that half of the patients do not express *MUTYH* at all in the tumor tissue, suggesting that maybe other currently undetectable mechanisms cause the complete loss of expression of this gene. Along this same line, we noted that the remaining patients showed only a partial expression of the protein, again indicating some sort of dysregulation of *MUTYH*, regardless of the presence of biallelic genomic variants.

Clinically, considering the immunohistochemistry and the genomic results, we suggest keeping more restricted surveillance on heterozygous carriers until the role of the monoallelic variants in *MUTYH* can be clarified.

## Figures and Tables

**Table 1:** Demographic and clinical characteristics of the patients enrolled in the present study. * In first- and second-degree relatives.

ID	Birth Date	Age at Cancer Diagnosis	Familiarity for Neoplasms *	% of Neoplastic Cells in the Tumor Sample	Diagnosis
pz 1	3 July 1943	76	positive	60	breast and other tumors
pz 2	30 June 1948	72	positive	30	ovarian carcinoma
pz 3	15 October 1979	37	positive	60	adenocarcinoma of endometrium extended to ovary
pz 4	6 January 1979	41	positive	30	breast carcinoma
pz 5	11 January 1970	44	positive	20	breast carcinoma
pz 6	24 March 1961	60	negative	80	adenocarcinoma of endometrium
pz 7	23 November 1975	42	positive	60	bilateral breast carcinoma
pz 8	8 December 1997	23	positive	40	bilateral ovarian carcinoma
pz 9	4 August 1966	55	positive	40	adenocarcinoma of endometrium

**Table 2:** (panel A) germinal variant of *MUTYH* (NM_001128425.2) detected in DNA extracted from peripheral blood samples and confirmed in the tumor sample; (panel B) somatic variants of *MUTYH* (NM_001128425.2) detected in tumor DNA sample.

**Panel A**						
**ID**	**Germinal Variant**	**Exon**	**ClinVar Class**	**AMCG Class**	**Variants in Other Genes**	
pz 1	c.1187 G>A p.Gly396Asp	13	LP/P	P	/	
pz 2	c.1187 G>A p.Gly396Asp	13	LP/P	P	BRCA1 VUS (c.5332 G>A, p. Asp1178Asn)	
pz 3	c. 536A>G p.Tyr179Cys	7	LP/P	P	/	
pz 4	c.1187 G>A p.Gly396Asp	13	LP/P	P	/	
pz 5	c. 536A>G p.Tyr179Cys	7	LP/P	P	BRCA2 VUS (c.3680 T>C p.Leu1227Pro) and CDH1 VUS (c.74C>T p. Pro25Leu)	
pz 6	c. 536A>G p.Tyr179Cys	7	LP/P	P	/	
pz 7	c.1187 G>A p.Gly396Asp	13	LP/P	P	PALB2 VUS (c.398 G>A p.Ser133Asn) and ATM VUS (c.1516 G>T p.Gly506Cys)	
pz 8	c.1258 C>A p.Leu420Met	13	CIP	VUS	/	
pz 8	c.694 A>T p.Thr232Ser (in cis with the other variant)	9	VUS	VUS	/	
pz 9	c.1437_1439del p.Glu480del	14	P	P	POLE VUS (c.6445 C>T p.Arg2149Cys)	
**Panel B**						
**ID**	**Variant**	**Exon**	**GnomAD Freq**	**ClinVar**	**ACMG Classification**	**In Silico Predictors**
pz 1	/	/	/	/	/	/
pz 2	/	/	/	/	/	/
pz 3	c.770 T>C p.Leu257Pro	9	absent	/	VUS	4VUS/35B
pz 3	c.1256 C>T p. Ala419Val	13	0.00003184	VUS	VUS	1P/6VUS/17B
pz 4	/	/	/	/	/	/
pz 5	c.312 C>T p.Tyr104Tyr	3	0.001488	CIP (1VUS/18LB/7B)	LB	2B
pz 6	/	/	/	/	/	/
pz 7	c.420 C>T p.Thr140Thr	5	absent	LB	LB	2B
pz 8	c.1014 G>C p.Glu338His	12	0.286	B	B	1VUS/29B
pz 9	c.1014 G>C p.Glu338His	12	0.286	B	B	1VUS/29B

CIP = conflicting interpretation of pathogenicity; LP = likely pathogenetic; P = pathogenetic; VUS = variant of unknown significance.

**Table 3:** Immunohistochemistry results. Score values refer to neoplastic cells only.

ID	SCORE 0	SCORE 1+	SCORE 2+	SCORE 3+	IHC Score
pz 1	60	20	10	10	70
pz 2	100	0	0	0	0
pz 3	50	30	10	10	80
pz 4	100	0	0	0	0
pz 5	100	0	0	0	0
pz 6	70	5	5	20	75
pz 7	20	5	15	60	215
pz 8	100	0	0	0	0
pz 9	60	10	10	20	90
control 1	100	0	0	0	0
control 2	100	0	0	0	0

## Data Availability

The original contributions presented in the study are included in the article, further inquiries can be directed to the corresponding author.

## Supplementary Materials

The following supporting information can be downloaded at https://www.mdpi.com/article/10.3390/genes15050554/s1, Supplementary Table S1: primer sequences used for PCR and Sanger sequencing. Supplementary Figure S1: *MUTYH* protein expression in non-neoplastic cells used as positive controls (lymphocytes and tonsillae tissues).
