# Supplementary material for: Exploring the Role of the MUTYH Gene in Breast, Ovarian and Endometrial Cancer
Source: Genes (Basel). 2024 Apr 26;15(5):554. doi: 10.3390/genes15050554 (PMC11120896; doi:10.3390/genes15050554)
Supplement: Supplementary file 1 [file genes-15-00554-s001.zip › genes-2972670-supplementary.pptx]

## Slide 1
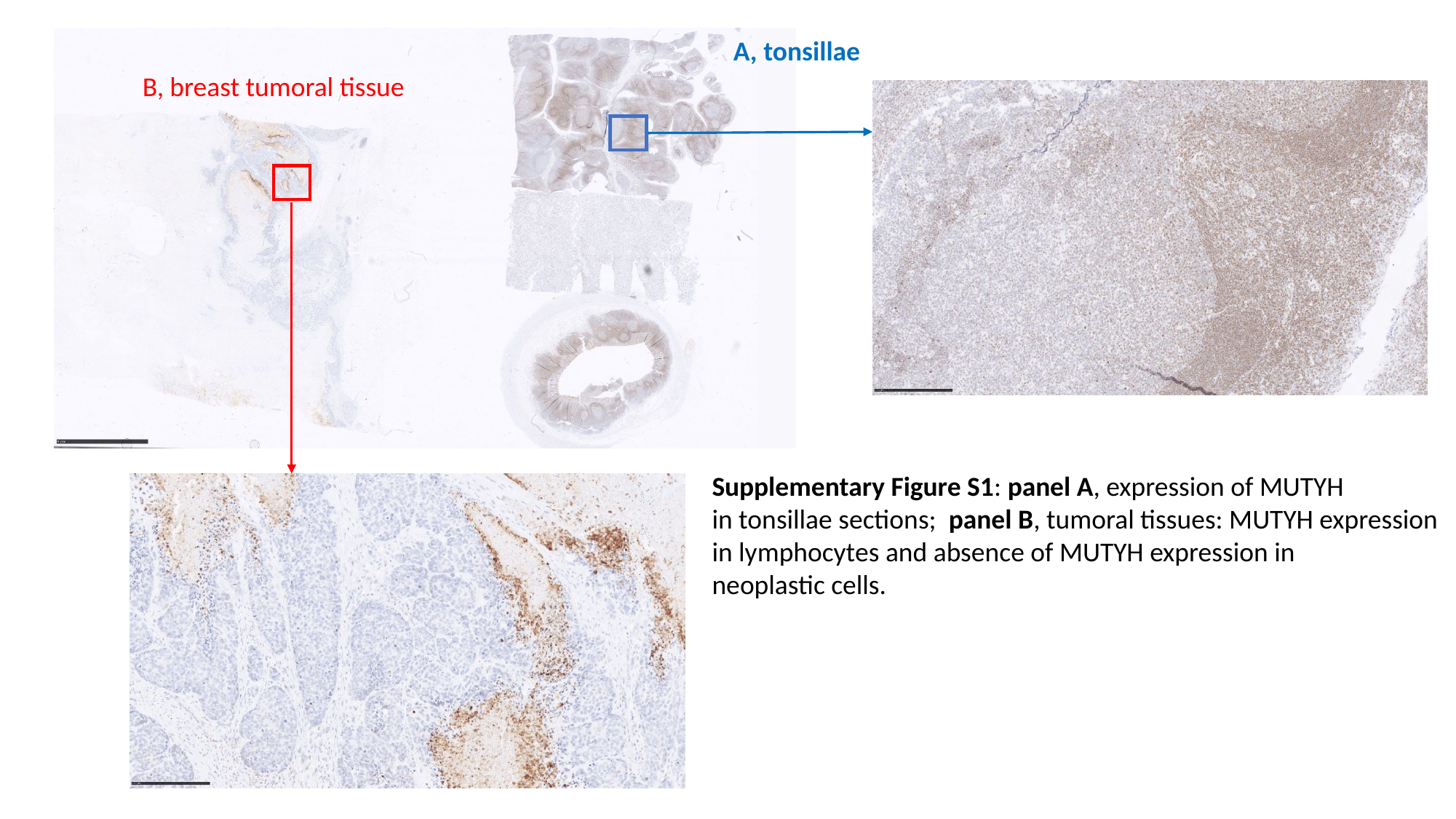

A, tonsillae
B, breast tumoral tissue
Supplementary Figure S1: panel A, expression of MUTYH
in tonsillae sections; panel B, tumoral tissues: MUTYH expression
in lymphocytes and absence of MUTYH expression in
neoplastic cells.
